# Comparison of HER2 expression between primary colorectal cancer and their corresponding metastases

**DOI:** 10.1002/cam4.228

**Published:** 2014-03-25

**Authors:** Won-Suk Lee, Yeon Ho Park, Jung Nam Lee, Jeong-Heum Baek, Tae-Hoon Lee, Seung Yeon Ha

**Affiliations:** 1 Department of Surgery, Gil Medical Center, Gachon University, School of MedicineIncheon, Korea; 2 Department of Pathology, Gil Medical Center, Gachon University, School of MedicineIncheon, Korea

**Keywords:** Colon cancer, HER2, KRAS, metastasis

## Abstract

The aim of this study was to compare human epidermal growth factor 2 (HER2) status in primary colorectal cancer and paired liver or lung metastasis. Gene amplification of HER2 has been intensively evaluated in contemporary oncology, especially in breast and stomach cancer. The knowledge of HER2 status in primary and metastatic sites may be of potential value for therapeutic decision making in metastatic colon cancer. The HER2 status was assessed by fluorescence in situ hybridization (FISH) and immunohistochemistry (IHC) in 94 colorectal cancer with corresponding liver or lung metastases. HER2 amplification was present in 19 of the 188 (10.1%) of both primary and metastases combined. Four (4.6%) patients showed HER2 amplification in the metastasis and 10 (10.6%) patients showed HER2 amplification in the primary tumor. In 14 cases (14.8%), the HER2 status of the primary lesions was different from that of the associated metastases. The presence of HER2 overexpression in *KRAS* mutant colon cancer was found in 5.3%. No relationship was found between HER2 expression and KRAS status (*P* = 0.486). The evidence of HER2 positive metastatic lesion and primary colorectal cancer suggest that HER2 assessment might be considered in selected cases when this may help change the therapeutic decision.

## Introduction

Colon cancer accounts for 10% of all new cancer diagnoses and 11% of all cancer-related deaths. It is also the fourth most common malignancy worldwide, with ∼1,000,000 new cases and 500,000 deaths recorded each year 1. Current estimates suggest that over 50% of patients with colon cancer will either have liver metastases at presentation or subsequently develop metastasis. Among patients who undergo curative resection for colon cancer, 10–20% will develop pulmonary metastasis and 10% of these patients will have isolated pulmonary lesions 2. Patients with curatively resected colon cancer and isolated liver or lung metastases have 5-year overall survival rates of 30–50% 3. Conventional chemotherapy for metastatic colon cancer with fluorouracil and leucovorin, possibly combined with irinotecan or oxaliplatin, may prolong progression-free and overall survival 4; however, long-term results have been less than satisfactory. As a result, there is extensive ongoing research on alternative therapeutic targets and agents.

It is currently unclear whether HER2 is a potential therapeutic target in patients with colon cancer, and HER2 is expressed at a far lower level in this malignancy than in breast cancer 5–7. In colon adenocarcinoma, reports of *HER2* gene amplification and the overexpression of HER2 protein have been inconsistent, with incidence rates ranging from 0% to 83% of primary tumors 8–10. Monoclonal antibodies such as cetuximab and panitumumab that target the epidermal growth factor receptor (EGFR) have proven to be efficacious in terms of response rate and progression-free survival when combined with standard cytotoxic chemotherapy for metastatic colon cancer 11–13. However, HER2-targeted therapy for colon cancer has not yet been studied.

Mutations in the v-Ki-ras2 Kirsten rat sarcoma viral oncogene homolog (*KRAS*), which occur most frequently in codons 12, 13, and 61, are found in ∼40% of colon tumors 14–16. *KRAS* mutations have emerged as a key negative predictive factor for treatment response in patients receiving cetuximab 16,17. These studies suggest that wild-type *KRAS* colorectal tumors would be responsive to cetuximab. However, it has not been established whether there is a relationship between *KRAS* and HER2 status.

HER2 status is usually evaluated in primary lesions because metastatic sites are rarely biopsied before the start of treatment. The aim of this study was to establish whether the HER2 status and *KRAS* status of primary tumors reflect that of their associated metastases and thus whether decisions to treat metastatic disease with anti-HER2 agents can be based on the assessment of the primary tumors. This is, to our knowledge, the first study to compare HER2 and *KRAS* status between primary tumors and the distant metastases that arise from them.

## Material and Methods

In this study, we evaluated 94 consecutive patients with colon cancer who underwent curative resection for primary and synchronous or metachronous liver or lung metastatic cancer. None of the patients were treated with trastuzumab-based chemotherapy.

### Specimens

All colon adenocarcinoma samples were obtained during operations performed at the Gachon University of Science and Medicine, Gil Hospital between January 2006 and March 2010. Among the 456 patients who underwent curative or palliative resection for colon cancer, 116 consecutive patients stage IV disease were randomly selected at the outpatient department for analysis. Complete clinical and pathological information was available for analysis in 94 of these cases. The tumor sections were first selected under the microscope to ensure that they comprised at least 70% neoplastic cells. Formalin-fixed, paraffin-embedded tissue blocks, selected on the basis of quality and representativeness of the sample, were cut into 5-*μ*m thick sections and examined using both fluorescent in situ hybridization (FISH) and immunohistochemistry (IHC). For each case, two tissue sections cut at different levels of the histological block were processed to control for tissue heterogeneity.

### IHC assessment of HER2 expression

Sections of archived formalin-fixed, paraffin-embedded tissue (3-*μ*m thick) were placed on slides coated with polylysine. After deparaffinization and blocking of endogenous peroxidase, HER2 immunostaining was performed using rabbit anti-human c-erbB-2 as a primary antibody (Dako Corp, Carpinteria, CA) at a 1:100 dilution. Primary antibody binding was assessed using the Dako Quick-Staining, Labeled Streptavidin–Biotin System (Dako), and this was followed by the addition of diaminobenzidine as a chromogen. HER2 immunoreactivity was evaluated by a single pathologist according to the scoring system described by Hofmann et al. 18. Resected samples exhibiting a strong (3+) complete, basolateral, or lateral membranous reactivity in ≥10% of cells were scored as positive. Samples with no reactivity, membranous reactivity in <10% of cells, or faint or barely perceptible membranous reactivity (1+) in ≥10% of tumor cells (i.e., cells for which only part of the membrane is reactive) were considered to be negative. Samples showing a weak to moderate complete, basolateral, or lateral membranous reactivity (2+) in ≥10% of tumor cells were scored as equivocal.

### FISH assessment of HER2 expression

Sections were dried at 60°C overnight, deparaffinized in xylene, and hydrated through a graded alcohol series to distilled water. The specimens were heated in a pretreatment solution (Dako, K5331) in a domestic microwave oven for 10 min, and then subjected to proteolytic digestion using pepsin (Dako, K5331) at room temperature for 10 min. Hybridization was performed in a hybridizer (Dako, S2450) at 82°C for 5 min, and then at 42°C for 16 h. The probes were based on the Probe Mix (Dako, K5331), containing a mixture of Texas red-labeled cosmid clones covering 220 kb of the HER2 amplicon and a mixture of fluorescein (fluorescein isothiocyanate)-labeled peptide nucleic acid probes targeted at the centromeric region of chromosome 17. After a stringent wash at 65°C for 10 min, the slides were mounted with a fluorescence mounting medium containing 4′,6-diamidino-2-phenylindol dihydrochloride and a coverslip was used. The slides were stored at 2–8°C in the dark until evaluation, which was performed within 2 weeks using a fluorescence microscope (DMRXA, Leica, Wetzlar, Germany).

### IHC assessment of EGFR expression

Immunohistochemical studies were performed on 5-*μ*m thick tissue microarray sections. The slides were heated at 60°C for 1 h and then rehydrated with 100% xylene (four washes for 3 min each), 100% ethanol (four washes for 3 min each), and running water (5 min). The sections were blocked for endogenous peroxidase activity with 1.5% hydrogen peroxidase in methanol (15 min) and then washed under running water for 5 min. This was followed by digestion with 0.01% bacterial protease type XXIV (Sigma Chemical, St Louis, MO) in prewarmed 5 mmol/L Tris buffer (pH 7.6) at 37°C for 10 min and washing under running water for 5 min. The sections were then transferred into Tris-buffered saline and incubated for 30 min with EGFR antibody clone H11 (Dako North America, Carpinteria, CA) at a 1:200 dilution and incubated in horseradish peroxidase-labeled polymer (Dako) for 30 min according to the manufacturer's instructions. The slides were washed with Tris-buffered saline between incubations. The tissue sections were stained using 3,3-diaminobenzidine as a chromogen (Dako) and counterstained with Mayer's hematoxylin. A glioma section was used as a positive control, and the negative control sections were incubated with negative control rabbit immunoglobulin (Dako) in the absence of primary antibody.

A modified EGFR expression scoring system was used, based on previously published criteria 14,15. Staining intensity was categorized as 0 (no staining), 1+ (weak membrane staining), 2+ (moderate, complete membrane staining), and 3+ (strong, complete membrane staining) 19.

### DNA extraction and KRAS mutation analysis

Hematoxylin/eosin-stained sections of 5-*μ*m thickness obtained from a representative paraffin-embedded block were placed on slides without coverslips for microdissection and DNA extraction. Briefly, microdissection was performed under direct observation using an inverted microscope and a sterile needle. Each microdissected sample was directly transferred to an Eppendorf tube that contained digestion buffer (2 mg/mL proteinase K in 50 mmol/L Tris [pH 8.5], 1 mmol/L ethylenediaminetetraacetic acid, 0.5% Tween 20). The tubes were then incubated overnight at 56°C, and this was followed by a 10-min incubation at 95°C to eliminate any remaining proteinase K activity. Polymerase chain reaction (PCR) was performed in 20-*μ*L reactions that contained 2 *μ*L of DNA, 2 *μ*L of commercial PCR buffer (Applied Biosystems, Foster City, CA), 2.0 mmol/L of MgCl_2_, 200 mmol/L of each deoxynucleotide triphosphate, 20 pmol of each primer, and three units of AmpliTaq Gold polymerase (Applied Biosystems). A Uno II Thermoblock (Biometra, Gottingen, Germany) was used for thermal cycling. Initial denaturation at 95°C for 10 min was followed by 41 cycles of denaturation at 95°C for 1 min, annealing at 52°C for 1 min, and extension at 72°C for 2 min and a final extension step at 72°C for 10 min. Exon 2 of the *KRAS* gene was amplified by PCR using intron-based primers in order to investigate the mutational status of *KRAS* codons 12 and 13, which occur frequently in colon cancer. The forward and reverse oligonucleotide primers used to amplify *KRAS* exon 2 were 5′-CAT GTT CTA ATA TAG TCA CA-3′ and 5′-AAC AAG ATT TAC CTC TAT TG-3′, respectively.

The amplified DNA was electrophoresed on a 2% agarose gel for 1 h at 110 V. The amplification products were then purified using a MinElute PCR purification Kit (Qiagen, Valencia, CA) according to the manufacturer's instructions. The PCR products were then sequenced in both directions using the ABI Prism BigDye Terminator v1.1 Cycle Sequencing kit (Applied Biosystems) and the same primers as those employed for PCR. The PCR products were finally purified on Centri-Sep Spin Columns (Applied Biosystems) and subsequently run on an ABI Prism 310 automatic sequencer (Applied Biosystems). The data were analyzed using Sequencing Analysis 5.2 Software (Applied Biosystems) 20.

### Statistics

Pearson's correlation test was used to compare the HER2 status of metastases assessed by IHC and FISH. The similarity in the HER2 IHC status between primary lesions and metastases was calculated as the ratio of concordant cases to total cases. The *κ*-coefficient was used to assess the level of agreement between samples, with *κ*-values between 0.61 and 0.8 considered to indicate a very good agreement. Differences were considered statistically significant when the *P*-value was ≤0.05. All statistical tests were two sided.

## Results

*HER2* gene copy number was evaluated using FISH in 94 consecutive primary colon adenocarcinomas and their matched liver or lung metastatic lesions (88 liver samples, six lung samples; Table 1). All samples were from a total of 94 matched surgical resections. The relevant patient characteristics are summarized in Table 1. A synchronous metastasis was detected in 66 patients (70.2%).

**Table 1:** Patient characteristics

	Number of patients (*n* = 94)	%
Age, range	60.8, 41–87	
Gender		
Male	66	70.2
Female	28	29.8
Primary tumor site		
Colon	65	69.0
Rectum	29	31.0
Primary tumor TN stage		
T1–T2	10	10.6
T3–T4	84	89.4
N0	21	22.3
N1	36	38.3
N2	37	39.4
Lymphovascular inv.		
Positive	67	67.0
Negative	33	33.0
Cell differentiation		
Well	37	39.4
Moderate	53	56.4
Poor/Mucinous	4	4.2
Synchronous mets	66	70.2
Metachronous mets	28	29.8
Metastatic sites		
Liver	88	93.6
Lung	6	6.4
Chemotherapy		
No chemotherapy	6	6.4
Adjuvant	81	86.2
Palliative	7	7.4

Among the metastatic lesions, *HER2* amplification was observed in 9 of the 94 (9.5%) histological specimens (Table 2) and in 19 of the 188 (10.1%) combined primary lesions and metastases. A cluster pattern of amplification was noted in the 14 *HER2*-positive metastases that were associated with synchronous metastasis (*P* = 0.033). In 14 cases (14.8%), the *HER2* status of the primary lesions was different from that of the associated metastases. Four (4.6%) patients showed *HER2* amplification in the metastasis but not in the primary sample, and 10 (10.6%) patients showed the opposite pattern, with *HER2* amplification in the primary tumor but not in the metastasis (Fig. S1). Figure S2 shows the patterns of *HER2* cluster amplification in primary tumors and matched liver metastases. Only five (5.5%) patients showed concordant HER2 expression in primary and metastatic sites.

**Table 2:** HER2 FISH on distant metastatic sites of CRC and matched primary tumors

	HER2 distant metastatic sites (*n* = 94)	
	
	FISH −	FISH +
HER2 primary site (*n* = 94)		
FISH −	75 (79.8%)	4 (4.2%)
FISH +	10 (10.6%)	5 (5.3%)

HER2 protein overexpression was assessed using IHC on histological sections obtained from 94 primary tumors and their matched 94 metastatic lesions obtained by surgical resection. Only 2.1% (2 of 94 cases) of the patients had HER2-positive tumors on IHC, with an immunopositive (3+) reaction in >80% of tumor cells. The total concordance between IHC and FISH was 86.1% (Table 3).

**Table 3:** HER2 status assessed by FISH and IHC in 94 primary CRC

	IHC score			
	
	Negative		Equivocal	Positive
			
	0	1+	2+	3+
FISH +	0	0	13	2
FISH −	64	15	0	0
Total, %	64 (68.1)	15 (16.0)	13 (13.8)	2 (2.1)

Of the 94 metastasis specimens assessed by IHC, 64 (68.1%) were negative for HER2 expression according to both IHC and FISH, two were positive according to both techniques, and 7 (7.4%) gave equivocal IHC results (Table 4). A comparison of HER2 protein expression between the 94 primary tumors and their paired metastatic sites showed that the overall concordance was 85.1% (Table 5). The four discordant cases according to IHC analysis gave similar results on using FISH. Among these cases, HER2 immunostaining was negative in the primary tumor and positive in the metastasis in two patients, and showed the opposite pattern in the other two patients.

**Table 4:** Comparison of HER2 status assessed by both IHC and FISH on 94 matched liver or lung metastatic sites

	IHC score			
	
	Negative		Equivocal	Positive
			
	0	1+	2+	3+
FISH +	0	0	7	2
FISH −	79	6	0	0
Total, %	79 (84.0)	6 (6.5)	7 (7.4)	2 (2.1)

**Table 5:** Comparison of HER2 status assessed by IHC on 94 primary and matched metastatic sites

	IHC metastatic site			
	
	Negative		Equivocal	Positive
			
	0	1	2	3
IHC primary site				
Negative				
0	59	2	1	2
1	12	2	1	0
Equivocal (2+)	6	2	5	0
Positive (3+)	2	0	0	0

Analysis of the *KRAS* mutation revealed that the same mutation was present in the primary tumor and the corresponding liver metastasis in 94 cases (87.2%; 95% confidence interval [CI] 93.6–98.2%). In 12 cases (12.8%; 95% CI 1.8–6.4%), of which six involved synchronous metastases at diagnosis and six showed metachronous metastases development, we found a discordance in *KRAS* mutation status between primary tumors and metastases. Five of these patients had a *KRAS* mutation in the primary tumor but not in the liver metastasis. In three cases, the *KRAS* mutation differed between the primary tumor and the metastases; one of these patients had a Gly13Asp *KRAS* mutation, whereas the liver metastasis had a Gly12Ser mutation (Table 6). Taken together, the observed discordance was clinically relevant in only 12 patients (12.8%; 95% CI 0.7–4.2%).

**Table 6:** Comparison of K-ras status on 94 primary and matched metastatic sites

	KRAS distant metastatic sites	
	
	Wild type	Mutant
KRAS primary site		
Wild type	51 (54.3)	7 (7.5)
Mutant	5 (5.2)	31 (33.0)

Ten patients (10.6%) had negative EGFR expression in the primary lesions but had at least 1 EGFR-positive metastatic site (Table S1). No relationship was found between HER2 expression and *KRAS* status (*P* = 0.486, Table 7).

**Table 7:** HER2 and KRAS status on primary and metastatic cancer combined (*n* = 188)

	KRAS wild type	KRAS mutant	*P*-value
HER2 FISH +	14 (7.5)	10 (5.3)	0.486
HER2 FISH −	100 (53.2)	64 (34.0)	

## Discussion

After its development as a therapeutic target for patients with breast cancer, HER2 has been evaluated as a target for patients with other tumor types. This includes metastatic gastric cancer, for which HER2-targeted therapy resulted in a 37% improvement in overall survival, leading to the approval of trastuzumab by the United States Food and Drug Administration for patients with HER2-positive metastatic lesions 21. HER2 is overexpressed in 25–35% of human breast cancers 22, but the level and incidence of HER2 overexpression in primary colon cancers appears to be different. Several studies evaluating HER2 expression in colon cancer reported considerably different overexpression rates, ranging from 0% to 83% 10,23. Furthermore, very little is known about the concordance of HER2 status between primary tumors and their associated metastases, and indeed this is, to the best of our knowledge, the first study on this subject.

Our findings suggest that there is in fact a high level of concordance between the results of IHC and FISH used to assess HER2 status in colon cancer. In a majority of cases, both primary lesions and their corresponding metastases showed the same level and pattern of HER2 expression, indicating that the regulation of HER2 is maintained during metastasis. However, because of the relatively small sample size in this study, we cannot draw any conclusions about the relative HER2 expression in synchronous and metachronous metastases.

Although we found that HER2 protein is present in colon cancer, only in a few cases was its expression strong enough to consider it as a potential therapeutic target (2+ and 3+). The *HER2* amplification status was evaluated by FISH in 94 paired primary and metastatic lesions, revealing a total of 19 amplified and 169 unamplified genes in both sites combined. *HER2* amplification was concordant in only 5% of the primary and metastatic lesions. Previous studies showed that, for metastatic breast cancer, the HER2 neutralizing antibody Herceptin® is only effective in the therapeutic range. In a study by Ramanathan et al. 24, Her-2/neu-positive patients with advanced colorectal cancer were treated with trastuzumab (Herceptin® and irinotecan). Of the 138 screened patients, HER2 overexpression was only detected in 11 (8%; 2+ in 5 and 3+ in six patients), and this resulted in premature termination of the study. It seems, therefore, that HER2 is unlikely to play a major role in colon cancer therapy. However, as ∼5% of all colon cancers do overexpress HER2 both in the primary lesion and the corresponding metastasis, HER2-targeted therapy with trastuzumab may still be a treatment option for ∼60,000 patients worldwide each year 25. Therefore, further investigation of regimens involving trastuzumab as part of a large multicenter, multinational trial is warranted.

A further noteworthy finding of this study is that *KRAS* mutations are frequently the same in primary lesions and their matched liver or lung metastasis, with a concordance rate of 87.2%. HER2 was overexpressed in colon tumors harboring *KRAS* mutations in 5.3% of cases (10 of 188 cases). This suggests that trastuzumab is a possible treatment option for patients with colon cancer and *KRAS* mutation.

Patients with a metastatic colon tumor that overexpresses HER2 (5%) may benefit from trastuzumab therapy. An understanding of the evolution of gene signatures in colon cancer together with molecular profiling may facilitate the identification of molecular subtypes that can predict which patients will respond favorably to trastuzumab therapy.

## Supporting Information

**Figure S1.** Colon cancer and matched metastatic sites with HER2 IHC staining. (A and B); both colonic adenocarcinoma (A) and its metastatic adenocarcinoma in liver (B) are positive for HER2 in their membrane and cytoplasm colon cancer cells. (C and D); the colonic adenocarcinoma shows positive staining for HER2 (C). However, the metastatic lesion in liver displays negative staining (D). 200×. **Figure S2.** The HER2 cluster amplification in colon and liver tissues (A and B). No HER2 cluster amplication is seen in C (colon) and D (liver). **Table S1.** Comparison of EGFR status assessed by IHC on 94 primary and matched metastatic sites.
