# Supplementary material for: Comparison of HER2 expression between primary colorectal cancer and their corresponding metastases
Source: Cancer Med. 2014 Mar 25;3(3):674–80. doi: 10.1002/cam4.228 (PMC4101759; doi:10.1002/cam4.228)

Supplemental Table

Table 1. Comparison of EGFR status assessed by IHC on 94 primary and matched metastatic sites.

|  | IHC metastatic site | | | |
| --- | --- | --- | --- | --- |
|  | 0 | 1+ | 2+ | 3+ |
| IHC primary site |  |  |  |  |
| 0 | 18 | 7 | 2 | 1 |
| 1 | 10 | 39 | 0 | 0 |
| 2 | 3 | 10 | 1 | 0 |
| 3 | 0 | 2 | 1 | 0 |

Supplemental Figure Legends

Figure 1. Colon cancer and matched metastatic sites with HER2 IHC staining. A & B; Both colonic adenocarcinoma (A) and its metastatic adenocarcinoma in liver (B) are positive for HER2 in their membrane and cytoplasm colon cancer cells. C & D; The colonic adenocarcinoma shows positive staining for HER2 (C). However the metastatic lesion in liver displays negative staining (D). X 200

Figure 2. The HER2 cluster amplification in colon and liver tissues(A and B). No HER2 cluster amplication is seen in C(colon) and D(liver).

Figure 1. Colon cancer and matched metastatic sites with HER2 IHC staining. A & B; Both colonic adenocarcinoma (A) and its metastatic adenocarcinoma in liver (B) are positive for HER2 in their membrane and cytoplasm colon cancer cells. C & D; The colonic adenocarcinoma shows positive staining for HER2 (C). However the metastatic lesion in liver displays negative staining (D).X 200

A.
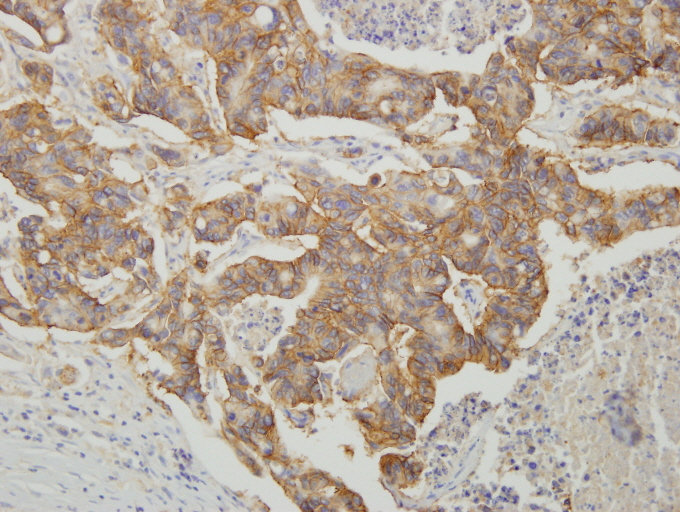


B.


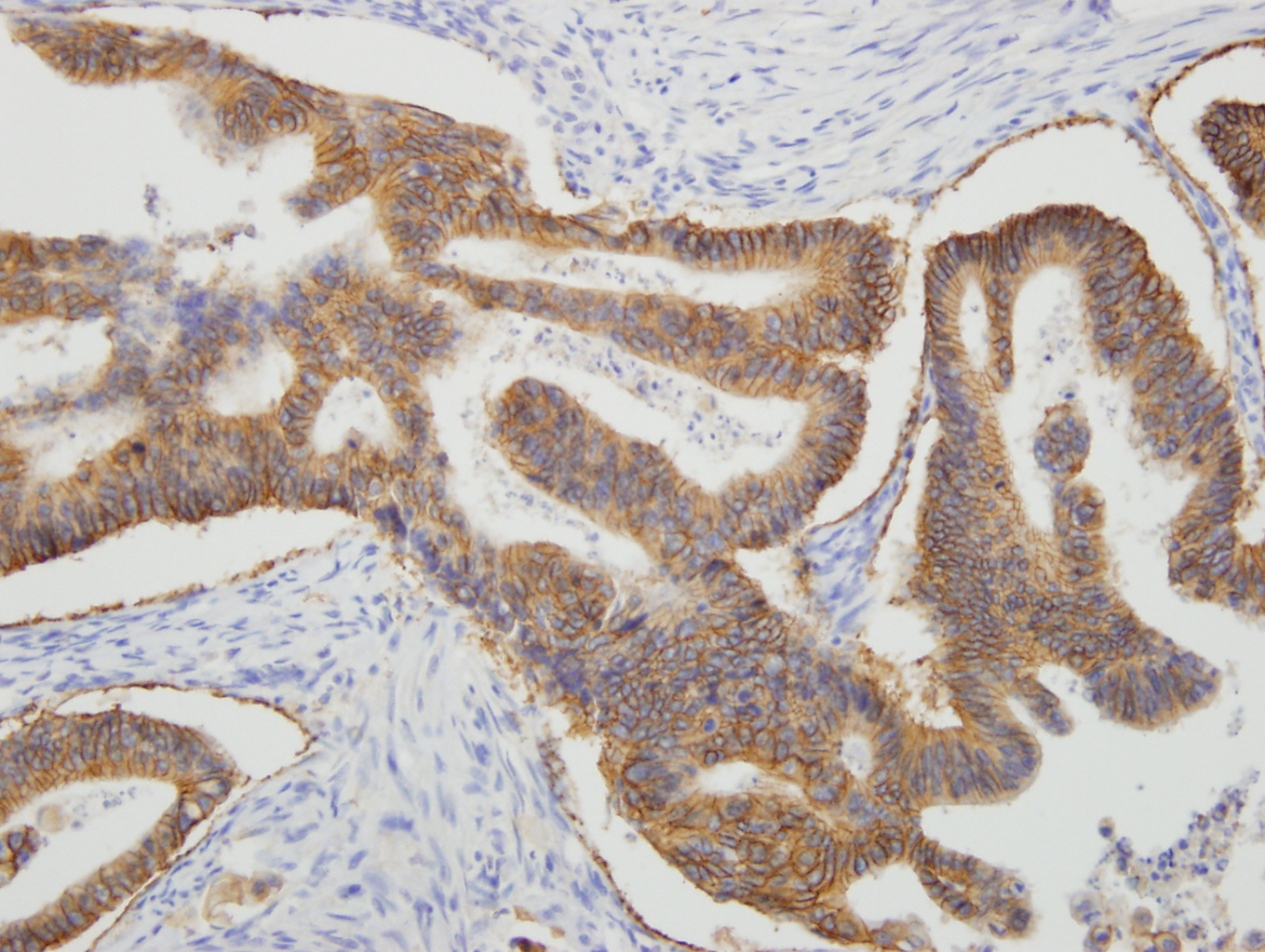


C.


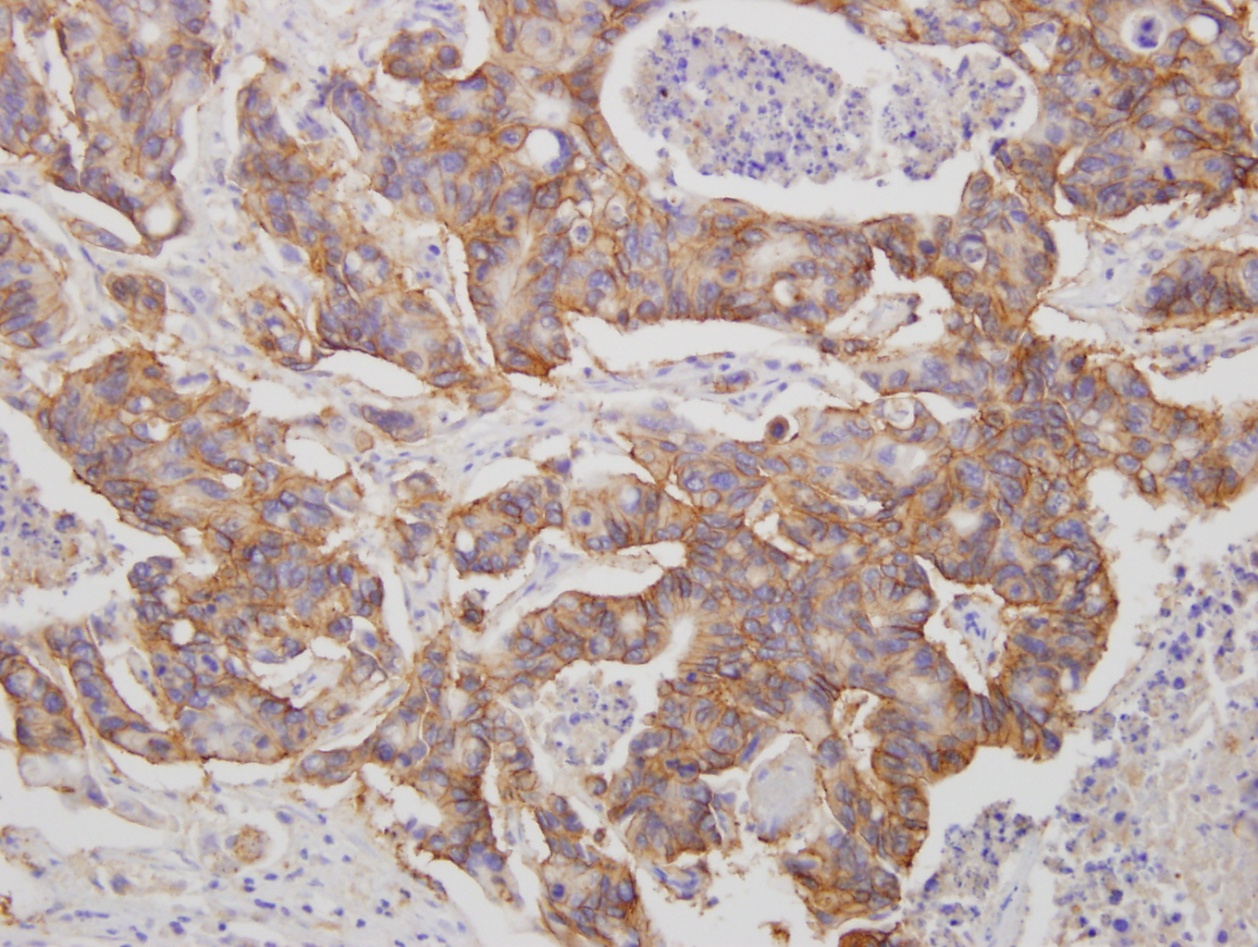


D.


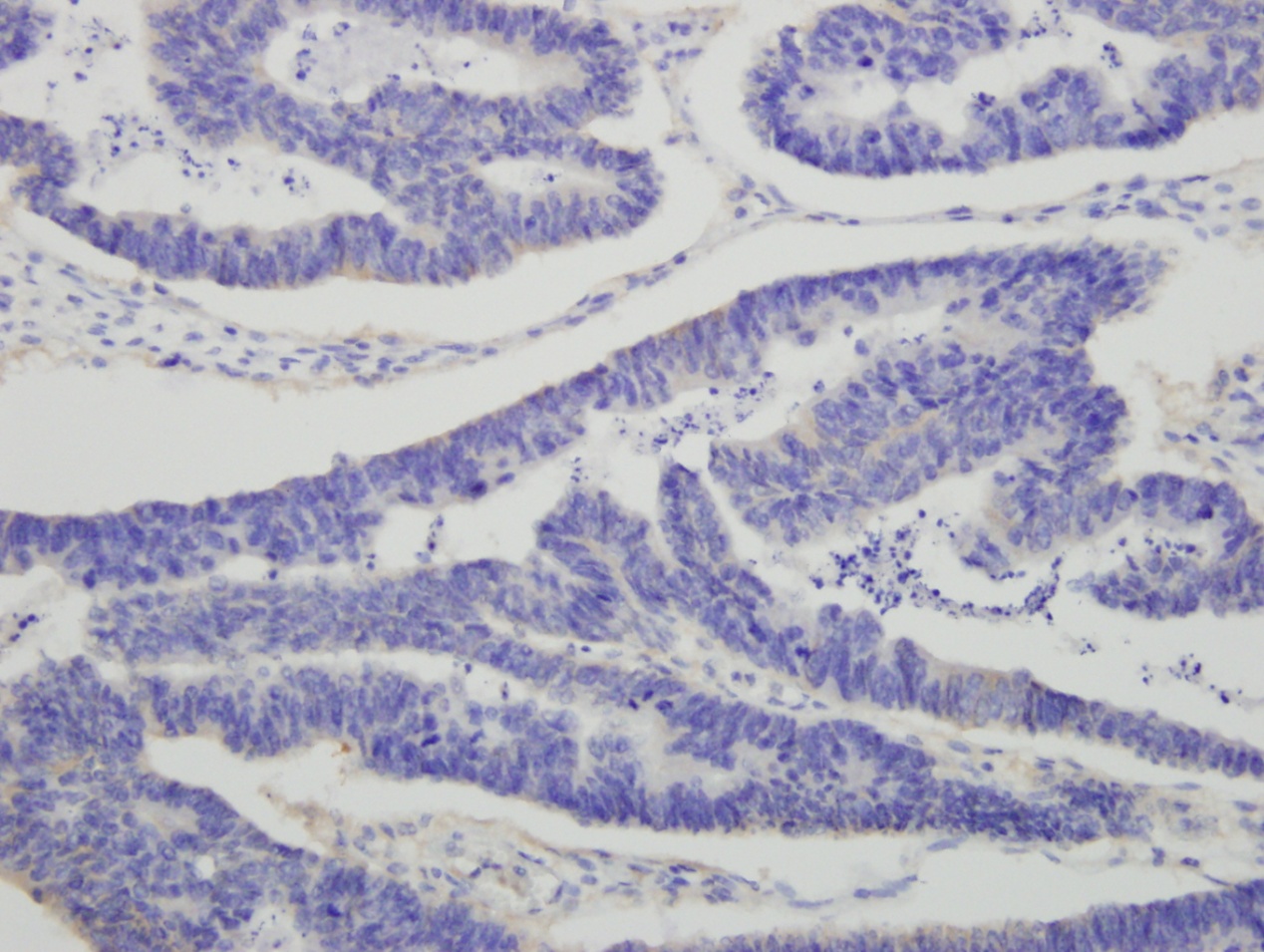


Figure 2. The HER2 cluster amplification in colon and liver tissues(A and B). No HER2 cluster amplication is seen in C(colon) and D(liver).

1. Colon, X1250
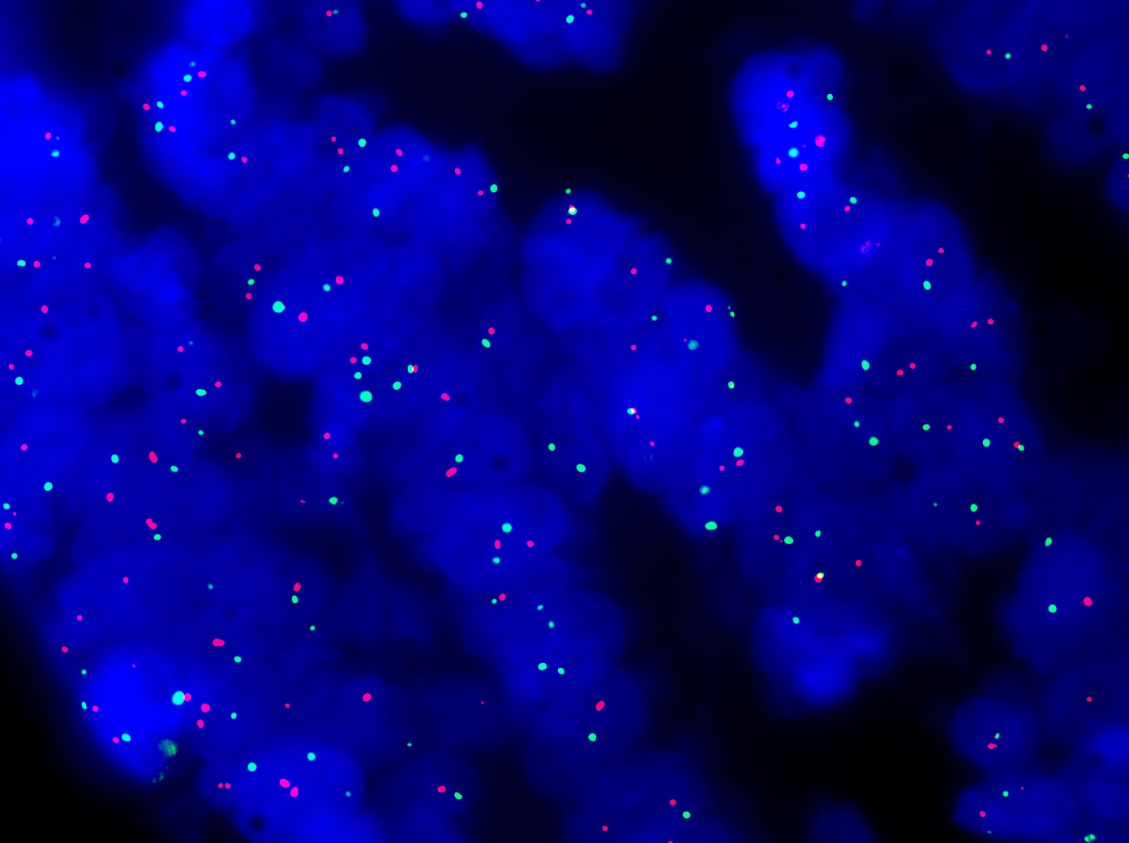

2. Liver, X1250


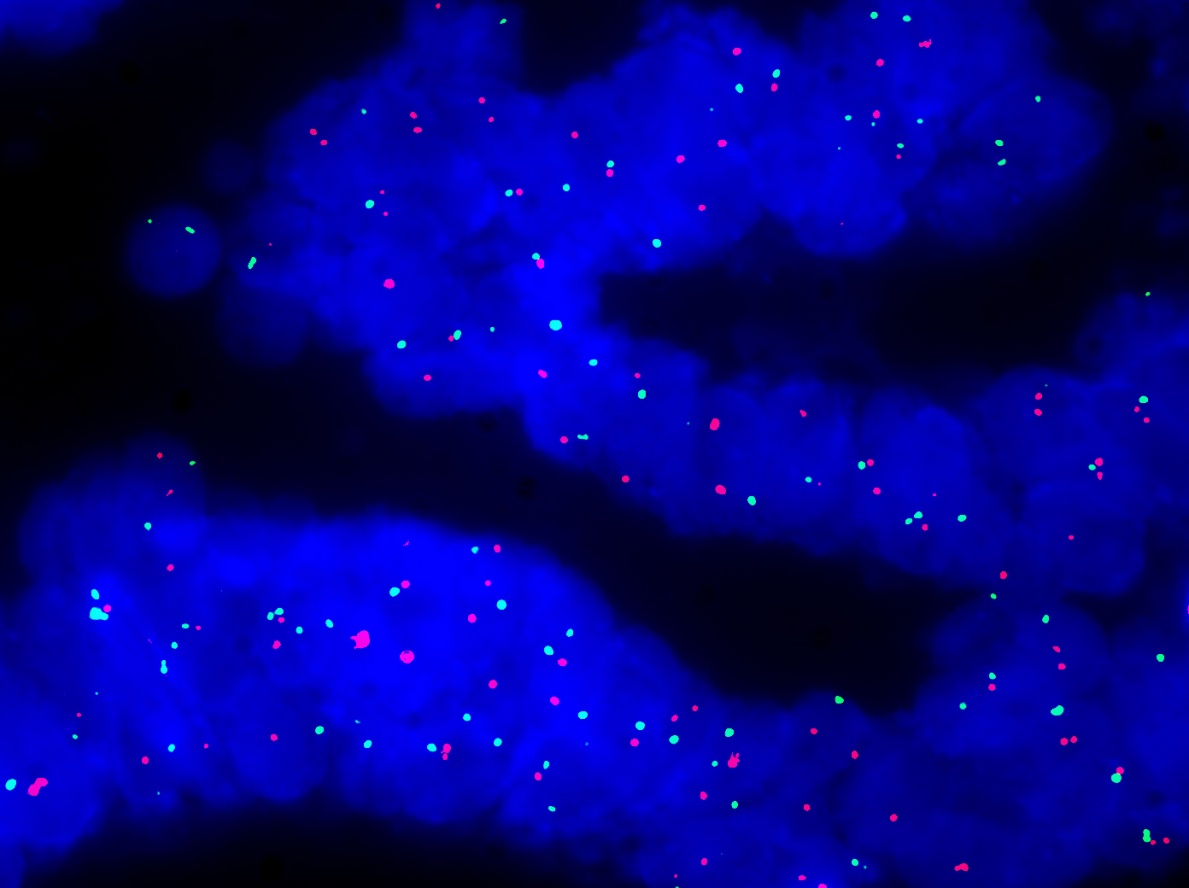


C. Colon, X1250


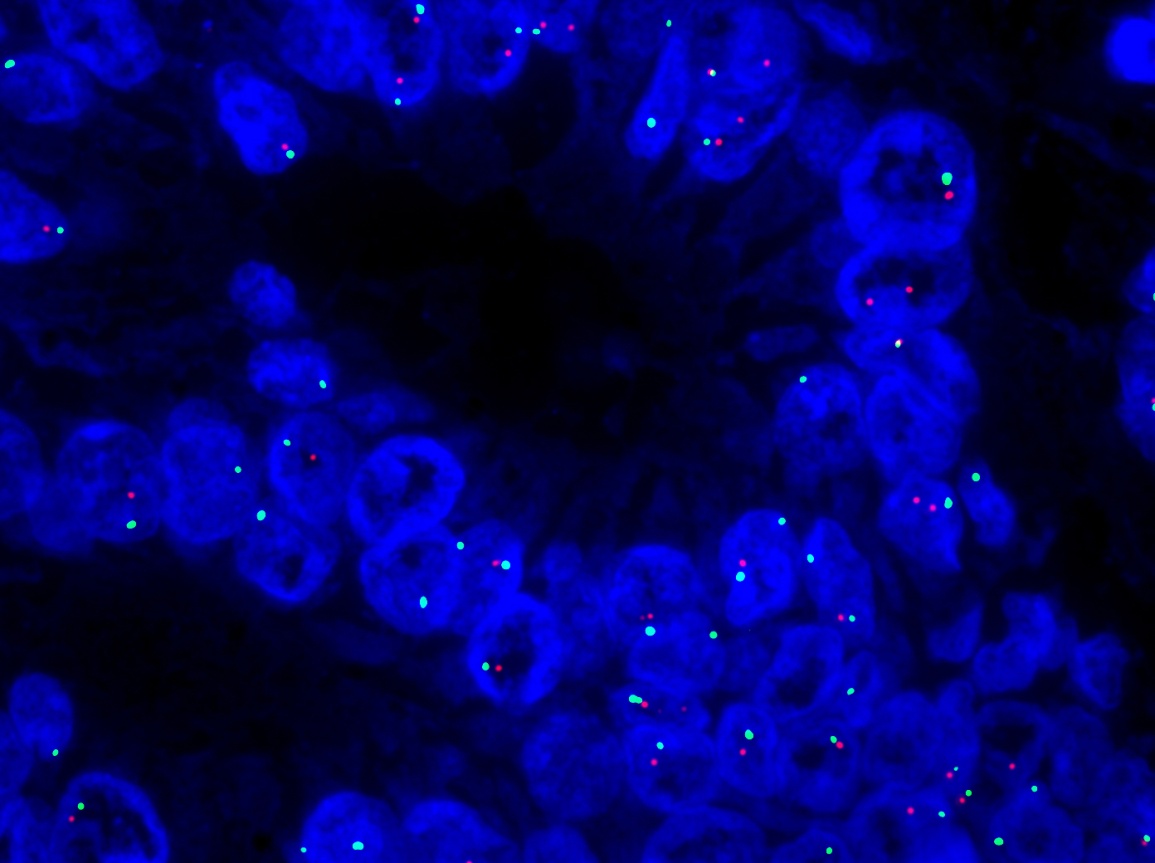

 D. Liver, X1250


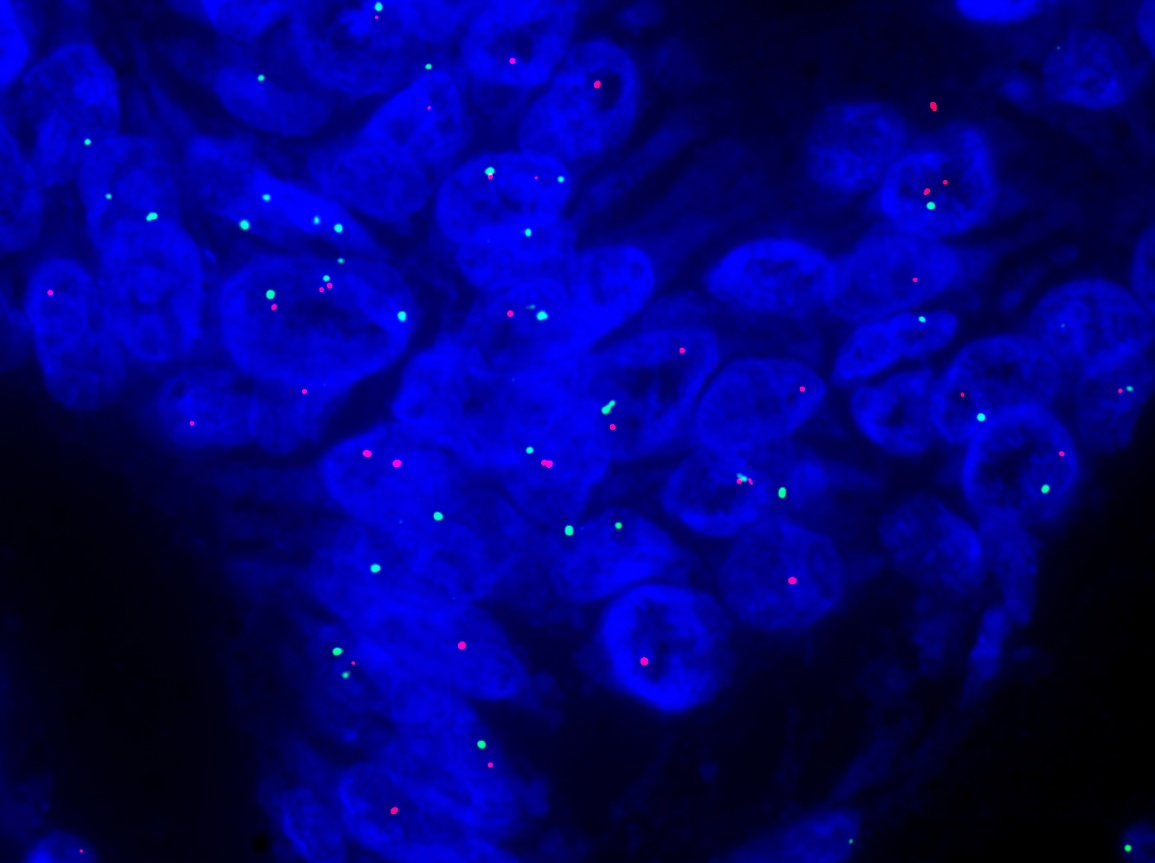

Supplement: Supplementary file 1 — Figure S1. Colon cancer and matched metastatic sites with HER2 IHC staining. (A and B); both colonic adenocarcinoma (A) and its metastatic adenocarcinoma in liver (B) are positive for HER2 in their membrane and cytoplasm colon cancer cells. (C and D); the colonic adenocarcinoma shows positive staining for HER2 (C). However, the metastatic lesion in liver displays negative staining (D). 200×. Figure S2. The HER2 cluster amplification in colon and liver tissues (A and B). No HER2 cluster amplication is seen in C (colon) and D (liver). Table S1. Comparison of EGFR status assessed by IHC on 94 primary and matched metastatic sites. [file cam40003-0674-SD1.docx]
